# Inflammation as a treatment target in mood disorders: review

**DOI:** 10.1192/bjo.2020.43

**Published:** 2020-06-05

**Authors:** Brett D. M. Jones, Zafiris J. Daskalakis, Andre F. Carvalho, Rebecca Strawbridge, Allan H. Young, Benoit H. Mulsant, M. Ishrat Husain

**Affiliations:** Centre for Addiction and Mental Health, Department of Psychiatry, University of Toronto, Canada; Centre for Addiction and Mental Health, Department of Psychiatry, University of Toronto, Canada; Centre for Addiction and Mental Health, Department of Psychiatry, University of Toronto, Canada; Department of Psychological Medicine, Institute of Psychiatry, Psychology & Neuroscience, King's College London, UK; Department of Psychological Medicine, Institute of Psychiatry, Psychology & Neuroscience, King's College London, UK; Centre for Addiction and Mental Health, Department of Psychiatry, University of Toronto, Canada; Centre for Addiction and Mental Health, Department of Psychiatry, University of Toronto, Canada

**Keywords:** Depressive disorders, bipolar affective disorders, neuroimmunology, antidepressants, novel CNS drugs

## Abstract

**Background:**

Mood disorders, i.e. major depressive disorder (MDD) and bipolar disorders, are leading sources of disability worldwide. Currently available treatments do not yield remission in approximately a third of patients with a mood disorder. This is in part because these treatments do not target a specific core pathology underlying these heterogeneous disorders. In recent years, abnormal inflammatory processes have been identified as putative pathophysiological mechanisms and treatment targets in mood disorders, particularly among individuals with treatment-resistant conditions.

**Aims:**

In this selective review, we aimed to summarise recent advances in the field of immunopsychiatry, including emerging pathophysiological models and findings from treatment ttrials of immunomodulatory agents for both MDD and bipolar disorders.

**Method:**

We performed a literature review by searching Medline for clinical trials of immunomodulating agents as monotherapy or adjunctive treatments in MDD and bipolar disorders. Included studies are randomised controlled trials (RCTs), cluster RCTs or cross-over trials of immunomodulating agents that had an active comparator or a placebo-arm.

**Results:**

Current evidence shows an association between inflammation and mood symptoms. However, there is conflicting evidence on whether this link is causal.

**Conclusions:**

Future studies should focus on identifying specific neurobiological underpinnings for the putative causal association between an activated inflammatory response and mood disorders. Results of these studies are needed before further treatment trials of immunomodulatory agents can be justified.

## Background

Major depressive disorder (MDD) and bipolar disorders are major sources of disability worldwide. It is estimated that approximately 350 million people experience MDD or bipolar disorders globally.^1^ According to the World Health Organization, MDD is the leading cause of disability worldwide, with the annual attributable financial loss of $83 billion.^2,3^

A diagnosis of MDD or bipolar disorder is made by conducting a psychiatric interview and assessing the presence and impact of psychopathological symptoms, while ruling out any medical conditions that may be directly causing these symptoms. Although some biomarkers associated with a mood disorder diagnosis have been identified, to date, none of these biomarkers have been adopted in clinical practice.^4^ In the absence of objective biomarkers to identify various disease states in psychiatry, treatment decisions are based largely on the patient's report of symptoms and a mental status examination.^5^

## Current pharmacological treatments for mood disorders

Pharmacological treatments for mood disorders were discovered serendipitously and they have remained fundamentally unchanged over the past several decades. Although antidepressant medications have evolved over the years, the therapeutic effect of all available antidepressants (with the possible exception of ketamine) is attributed to their action on at least one of the monoamine neurotransmitters.^6^ Pharmacotherapy for acute bipolar depression comprises medications used primarily for the treatment of mania (i.e. lithium, atypical antipsychotics), MDD (i.e. antidepressants) or the maintenance phase of bipolar disorders (for example lamotrigine). Although these medications are effective for a significant proportion of patients, large studies suggest that up to 50% of patients do not achieve remission with standard treatments.^7–9^ A possible explanation for this variability in treatment outcomes is that patients with mood disorders are a heterogeneous group. To achieve higher rates of remission, new studies could use the framework of ‘precision medicine’ and delineate subgroups of patients who are more likely to respond to specific treatments.

## Immunopsychiatry

In recent years, several pre-clinical and clinical studies have investigated the association between the inflammatory response system and neuropsychiatric disorders, including MDD and bipolar disorders.^10–17^ The subspecialty of ‘immunopsychiatry’, which aims to study this association, is now an established area of research. In this selective review we will summarise the evidence of the putative pathophysiological association between the inflammatory response system and mood disorders. We will also provide an updated summary of findings from treatment trials of immunomodulating agents in patients with MDD and bipolar disorders.

## Method

The literature was reviewed by searching Medline for clinical trials of immunomodulating agents as monotherapy or adjunctive treatments in MDD and bipolar disorders from inception until December 2019, as well as by reviewing relevant reviews and references in the field. Selected studies were randomised controlled trials (RCTs), cluster RCTs or cross-over trials of immunomodulating agents that had an active comparator or a placebo-arm. Participants had to meet criteria for MDD or bipolar disorders according to ICD-10, DSM-IV or DSM-5.^18–20^ We only reviewed studies in English.

## Results

### Are mood disorders associated with a proinflammatory state?

The immune system and its possible association with mental disorders has been studied since the late 1800s. In 1887, Julius Wagner-Jauregg induced malaria in patients to alleviate symptoms of ‘dementia paralytica’, later known to be the neuropsychiatric manifestations of syphilis.^21^ Almost a century later, Benjamin Hart coined the term ‘sickness behaviour’ to characterise the psychological and behavioural symptoms of an acute physical illness.^22^ In the early 1990s, the ‘macrophage theory’ of depression postulated that activated macrophages secrete proinflammatory cytokines that either precipitate or exacerbate a moodstate.^23^ Since then, numerous studies have linked the inflammatory response system to both MDD and bipolar disorders. Interest in this line of research has been fostered by the observation that nearly all chronic and autoimmune disorders are associated with a high comorbidity of depressive symptoms. For example, more than half of patients with rheumatoid arthritis or systemic lupus erythematosus experience depressive symptoms.^24^ Similarly, patients with Crohn disease and comorbid depression experience exacerbations of co-occurring physical and depressive symptoms.^25^ In a recent meta-analysis of anti-cytokine agents in autoimmune disorders, these medications were reported to lead to a significant improvement of depressive symptoms.^26^ Although the effect size for these medications is small to moderate, it is comparable with the effect size observed with traditional antidepressant medications.^27,28^

During the past decade, many cross-sectional observational studies have investigated peripheral inflammatory biomarkers in MDD. A meta-analysis of 82 such studies comprising over 3000 patients reported that levels of interleukin (IL)-6, IL-10, Il-12, IL-13, IL-18, the soluble IL-2 receptor (sIL-2R), tumour necrosis factor – alpha (TNF-α), the soluble TNF receptor 2 (sTNFR2), and chemokine ligand 2 (CCL-2) are significantly elevated in individuals with MDD compared with healthy controls.^29^ In another recent meta-analysis of 37 studies comprising 13 541 patients with depression and 155 728 controls, half of those with depression showed low-grade inflammation as evidenced by a C-reactive protein (CRP) level >1 mg/L, and a quarter had a CRP level ≥3 mg/L.^30^

Other meta-analyses have shown that traditional antidepressants (for example selective serotonin reuptake inhibitors (SSRIs) and serotonin–noradrenaline reuptake inhibitors) are associated with a reduction in IL-6, TNF-α and CCL-2^31^ and that persistently elevated TNF-α is associated with treatment resistance.^32,33^ Retrospective cohort studies indicate that inflammation may play a role in the onset of mood symptoms and that elevated peripheral inflammatory markers in early life are predictive of adult depressive symptomology.^34,35^

Within the literature, there is significant heterogeneity between studies and in particular, there are substantial differences in how comorbidities are controlled for. This is particularly important as protein-based inflammatory markers such as CRP fluctuate and are influenced by multiple factors including body mass index (BMI), medications, exercise, diet and substance use; all of which are difficult to account for and poorly reported in studies.^36^ Furthermore, there is evidence that IL-6 and CRP operate through multiple physiological pathways, which are not solely upregulated during the inflammatory response.^37^ In addition, CRP has a second isoform that is produced in the absence of inflammation and is postulated to have a net anti-inflammatory effect.^37^ Notwithstanding these limitations, current evidence supports an association between abnormal inflammatory processes and depressed mood, in at least a subset of individuals.

Much of the evidence in patients with bipolar disorders has been based on cross-sectional studies indicating that an activated inflammatory response may be associated with a mood state.^38^ As with MDD, several immune-related disorders, such as autoimmune disorders, obesity, type 2 diabetes and cardiovascular disease, have a higher rate of incidence in patients with bipolar disorders than in the general population.^39^ Patients with bipolar disorders are also at higher risk for inflammation-associated metabolic syndromes such as myocardial infarction, stroke, atherosclerosis and hypertension.^11,40–43^

Examination of post-mortem brain tissue can provide valuable insights into a possible association between mood disorders and neuroinflammation. In a meta-analysis of post-mortem studies of brains of patients with MDD measuring cytokines, chemokines and cell-specific markers of microglia and astrocytes, two studies found increased markers of microglia in MDD and four studies found no differences between MDD and healthy controls.^44^ Another similar meta-analysis had inconsistent results.^45^ Several of the 51 studies included in the meta-analysis showed evidence of inflammation in post-mortem brain samples of patients with bipolar disorders. However, these 51 studies evaluated different biomarkers of neuroinflammation: presence of infiltrating peripheral immune cells in the central nervous system, cytokines levels or microglia activation and very few evaluated these different biomarkers in the same post-mortem brain sample. Eight of 15 studies in the meta-analysis found no effect of bipolar disorders on microglia cell markers; 9 of 17 studied did not find any effect of bipolar disorders on astrocyte cells, whereas eight found a decrease, and two reported both an increase and a decrease in different brain regions.^45^ Given the heterogeneity of both the methods and results of these post-mortem studies, one cannot reach a conclusion from them whether there is a causal link between neuroinflammation and mood disorders. Future studies are needed to address this heterogeneity in the current post-mortem literature.

Given the limitations of post-mortem studies in mood disorders, the measurement of in-vivo indices related to neuroinflammation using brain imaging can provide additional evidence for a possible association between abnormal inflammatory processes and mood symptoms. In recent years, positron emission tomography (PET) has been used to evaluate glial cells in MDD. Translocator protein-18 kDa (TSPO) volume distribution (V_T_) is used as a measure of microglial activation because it is elevated in microglia that have morphological features of being activated. A PET study quantifying TSPO V_T_ with an early TSPO radiotracer – [^11^C]PK11195 – showed increased microglial activation in patients with bipolar disorders (*n* = 14) compared with healthy controls (*n* = 11).^46^

To our knowledge, no other published PET studies have investigated neuroinflammation in bipolar disorders. In a meta-analysis of PET studies in MDD, TSPO V_T_ was elevated in patients with MDD compared with controls in the anterior cingulate cortex (standardised mean difference (SMD) = 0.78, 95% CI 0.41–1.16) and temporal cortex (SMD = 0.52, 95% CI 0.19–0.85). However, recent evidence shows that TSPO displays incomplete specificity for microglia, and hence may be an unreliable radiotracer of neuroinflammation.^47^ Future PET studies of central inflammation in mood disorders should use more sensitive radiotracers.

### Putative pathophysiological models linking inflammation to MDD and bipolar disorder

**Fig. 1:**
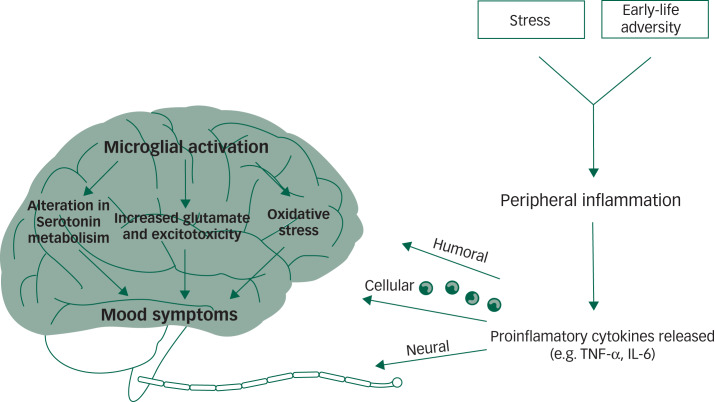
Inflammation and mood disorders.

Mechanistic studies are yet to confirm how inflammation may induce a mood episode in a subset of individuals. However, current theories on the ‘inflammatory model’ of depression postulate that in a subset of individuals with depression, psychosocial stress leads to the activation of the sympathetic nervous system and subsequent release of catecholamines (for example, norepinephrine), which stimulates bone marrow production and the release of myeloid cells (for example, monocytes) into the periphery. Monocytes are the main producers of inflammatory cytokines; once they enter the periphery, it is hypothesised that they can encounter stress-induced damage-associated molecular patterns (DAMPs) and microbial-associated molecular patterns (MAMPs) leaked from the gut, for example bacteria and bacterial products.^48^ These DAMPs and MAMPs then lead to the activation of inflammatory signalling pathways and the release of proinflammatory cytokines including TNF and IL-6, which can cross the blood–brain barrier through cellular, humoral and neural routes^48^ (see Fig. 1).

This figure has been simplified to focus on the main putative associations between inflammation and mood disorders. Chronic stress and early-life adversity can lead to persistent activation of the inflammatory response system. Proinflammatory cytokines released by an activated immune response can reach the brain via three main routes: humoral, cellular and neural. These cytokines lead to alterations in serotonin metabolism, increased glutamate and oxidative stress. The downstream effect of these alterations in neural circuits may lead to the onset and persistence of mood symptoms. IL, interleukin; TNF, tumour necrosis factor.

In the brain, the activated central inflammatory response influences neurotransmitter systems via metabolic or molecular pathways and by increasing the expression and functioning of presynaptic reuptake pumps.^48,49^ Several cytokines activate indoleamine 2,3-dioxygenase, which breaks down tryptophan, the primary precursor of serotonin, into kynurenine.^50,51^ This shunting of the production of serotonin towards kynurenine, combined with increased reuptake from the presynaptic serotonin pump, can lead to serotonin depletion. Furthermore, the kynurenine pathway is hypothesised to disrupt glutamate metabolism, leading to reduce astrocytic glutamate reuptake and stimulation of astrocyte glutamate release, leading to excess glutamate^52,53^ and reduced brain-derived neurotrophic factor (BDNF).^54^ Inflammatory effects on growth factors such as BDNF in the dentate gyrus of the hippocampus may affect learning and memory in patients with mood disorders.^48^

Proinflammatory cytokine effects on dopamine can also inhibit several aspects of reward motivation in corticostriatal circuits involving the basal ganglia, ventromedial prefrontal cortex and subgenual and dorsal anterior cingulate cortex, while also activating circuits in the amygdala and hippocampus.^48^ These physiological changes may present clinically as decreased motivation (anhedonia), avoidance, arousal, fear and alarm (anxiety) – core symptoms of depression and significant perpetuating factors for a depressive mood state.^48^

Although there are fewer studies examining putative associations between abnormal inflammatory processes and bipolar disorders, similar pathways may be involved. These include alterations in dopamine and glutamate metabolism and changes in proinflammatory cytokines, potentially leading to mitochondrial dysfunction and consequent increase in apoptosis, cell membrane damage and protein aggregation.^55^ In the context of bipolar disorders, increased dopamine release is postulated to lead to the formation of free radicals, causing oxidative damage.^56^ Mitochondrial electron transport chain dysfunction may in turn produce more free radicals.^57^ Activation of a proinflammatory cytokine receptor, such as TNF-α and IL-6 can lead to apoptosis through caspase activation and nitric oxide production.^55^

Glutamate may also cause excitotoxic damage in bipolar disorders through activation of the *N*-Methyl-d-aspartate receptor, which increases calcium influx and consequent nitric oxide production, leading to nitrosative damage to DNA, proteins and lipids.^55,58^ Oxidative and nitrosative damage can induce cell membrane damage, protein aggregation and apoptosis. This cascade has been hypothesised to lead to changes that result in the manifestation of depressive, manic and mixed episodes in bipolar disorders.^55^

### Use of immunomodulatory agents for the treatment of MDD and bipolar disorder

Given the prevalence of treatment-resistant depression in patients with MDD or bipolar disorders, it is likely that a subset of these patients do not respond to current standard antidepressant treatments because their treatment does not engage the appropriate neurobiological target. Conversely, if an activated inflammatory response is associated with MDD and bipolar disorders, therapeutic interventions targeting this pathophysiological process may confer benefit, at least in a subset of individuals.

In recent years, several studies have assessed the effect of immunomodulatory agents in neuropsychiatric disorders including MDD and bipolar disorders. These studies have most commonly evaluated non-steroidal anti-inflammatory drugs (NSAIDS), cytokine inhibitors and pleiotropic agents such as minocycline or *N*-acetylcysteine (NAC). Herein, we review evidence from selected clinical trials of these agents in MDD and bipolar disorders. Tables 1 and 2 outline the studies included.

**Table 1:** Identified clinical trials of anti-inflammatory agents in major depressive disorder (MDD)

Reference	Number of patients		Comorbidity/ enriched sample	Diagnosis	Treatment arms	Duration	Results	
	Randomised	Analysed					Active	Placebo
NSAIDs								
Müller et al (2006)^59^	40	18	None/No	MDD, DSM-IV	NARI + celecoxib 400 mg o.d. (*n* = 20) *v*. NARI + placebo o.d. (*n* = 20)	6 weeks	Final HRSD: 7.9, s.d. = 7.1	Final HRSD: 12.1, s.d. = 8.3
Akhondzadeh et al (2009)^60^	40	37	None/No	MDD, DSM-IV	SSRI + celecoxib 200 mg b.i.d. (*n* = 20) *v*. SSRI + placebo b.i.d. (*n* = 20)	6 weeks	HRSD change: –13.2, s.d. = 4.26	HRSD change: –10.2, s.d. = 3.77
Abbasi et al (2012)^62^	40	37	None/No	MDD, DSM-IV	SSRI + celecoxib 200 mg b.i.d. (*n*=15) *v*. SSRI + placebo b.i.d. (*n =* 15)	6 weeks	HRSD change: –13.4, s.d. = 3.88	HRSD change: –10.5, s.d. = 3.15
Majd et al (2015)^61^	30	23	None/No	MDD, DSM-IV	SSRI + celecoxib 100 mg b.i.d. (*n =* 20) *v*. SSRI + placebo b.i.d. (*n =* 20)	8 weeks	Final HRSD: 7.9, s.d. = 4.0	Final HRSD: 10.4, s.d. = 3.0
Cytokine inhibitors								
Raison et al (2013)^64^	60	60	None/No	MDD	Three infusions at week 0, 2, 6 of infliximab 5 mg/kg (*n =* 30) *v*. placebo (*n =* 30)	12 weeks	HRSD change: –7.6, s.d. = 7.0	HRSD change: –9.6, s.d. = 7.0
Minocycline								
Emadi-Kouchak et al (2016)^66^	50	46	HIV/No	MDD, HRSD <18	Minocycline 100 mg b.i.d.^23^ *v*. placebo b.i.d.^23^	6 weeks	HRSD change: –3.83, s.d. = 1.92	HRSD change: –1.65, s.d. = 2.12
Husain et al (2017)^68^	41	41	None/No	MDD, DSM-V	TAU+ Minocycline 200 mg o.d. (*n =* 21) *v*. TAU + placebo o.d. (*n =* 20)	12 weeks	HRSD final score: 15.1, s.d. = 13.2	HRSD final score: 32.0, s.d. = 11.8
Dean et al (2017)^69^	71	71	None/No	MDD, DSM-IV	TAU + Minocycline 200 mg o.d. (*n =* 36) *v*. TAU + placebo o.d. (*n =* 35)	12 weeks	Final MADRS: 15.8, s.d. = 10.6	Final MADRS: 19.8, s.d. = 9.8
*N*-Acetylcysteine								
Berk et al (2014)^72^	269	207	None/No	MDD, DSM-IV	TAU + NAC 500 mg b.i.d. (*n =* 135) *v*. TAU + placebo b.i.d. (*n =* 134)	12 weeks	MADRS change: –12.2, s.e. = 0.9	MADRS change: –10.7, s.e. = 1
Porcu et al (2018)^73^	NR	57	None/No	MDD/bipolar disorders, DSM-IV	CRP > 3: TAU + NAC 1.8 g o.d. (*n =* 15) *v*. TAU + placebo o.d. (*n =* 12) CRP < 3: TAU + NAC 1.8 g o.d. (*n =* 10) *v*. TAU + placebo o.d. (*n =* 30)	12 weeks	CRP > 3: Final HDRS: 5.20, s.d. = 6.53 CRP < 3: Final HDRS: 4.67, s.d. = 4.33	

NSAIDs, non-steroidal anti-inflammatory drugs; NARI, selective noradrenaline reuptake inhibitor; o.d., once daily; HRSD, Hamilton Rating Scale for Depression; SSRI, selective serotonin reuptake inhibitor; b.i.d., twice daily; TAU, treatment as usual; MADRS,  Montgomery–Åsberg Depression Rating Scale; CRP,  C-reactive protein; NR, not reported.

**Table 2:** Identified clinical trials of anti-inflammatory agents in bipolar disorders

Reference	Number of patients		Comorbidity/ enriched sample	Diagnosis	Treatment arms	Duration	Results	
	Randomised	Analysed					Active	Placebo
Cytokine inhibitors								
McIntyre et al (2019)^89^	60	58	None/Yes	Bipolar disorders (1 or 2) current depressed episode, DSM-5	Three infusions at week 0, 2, 6 of infliximab 5 mg/kg (*n =* 29) *v*. placebo (*n =* 31)	12 weeks	MADRS LSM change: –10.97	MADRS LSM change: –11.73
Minocycline								
Savitz et al (2018)^83^	99	95	None/No	Bipolar disorders (1, 2, or NOS, current MDE), DSM-IV	TAU + minocycline 100 mg b.i.d. + ASA 81 mg b.i.d. (*n =* 31) *v*. TAU + minocycline 100 mg b.i.d. + placebo (*n =* 19) *v*. TAU + ASA 81 mg b.i.d. + placebo b.i.d. (*n =* 19) *v*. TAU + placebo b.i.d. (*n =* 30)	6 weeks	Final MADRS: minocycline + ASA: 14.5, s.d. = 8.9 minocycline + placebo:15.5, s.d. = 8 ASA + placebo:12.3, s.d. = 8.4	Final MADRS: 16.0, s.d. = 9.6
*N*-acetylcysteine (NAC)								
Berk et al (2008)^85^	75	75	None/No	Bipolar disorders (Stable bipolar disorders 1 or 2)	TAU + NAC 1 g b.i.d. (*n =* 38) *v*. TAU + placebo b.i.d. (*n =* 37)	24 weeks	Final MADRS: 6.6, s.d. = 7.4	Final MADRS: 14.0, s.d. = 11.5
Bauer et al (201)^88^	38	24;20	None/No	Bipolar disorders (1 or 2 experiencing MDE or mixed episode), DSM-IV	TAU + ASA 500 mg b.i.d. *v*. TAU + NAC 500 mg b.i.d. *v*. TAU + ASA 500 mg b.i.d. + NAC 500 mg b.i.d. *v*. TAU + placebo b.i.d.	16 weeks	Final MADRS: NAC: week 8: 10.13, s.d. = 9.78; week 16: 11.38, s.d. = 10.36 ASA: week 8: 12, s.d. = 11.34; week16: 7.25, s.d. = 5.91 NAC+ ASA: week 8: 7.5, s.d. = 10.46; week 16: 5, s.d. = 6.25	Final MADRS: week 8: 7.13, s.d. = 8.29; week 16: 13.6, s.d. = 13.83
Porcu et al (2018)^73^	NR	57	None/No	MDD/bipolar disorders, DSM-IV	CRP > 3: TAU + NAC 1.8g o.d. (*n =* 15) *v*. TAU + placebo o.d. (*n =* 12) CRP < 3: TAU + NAC 1.8 g o.d. (*n =* 10) *v*. TAU + placebo o.d. (*n =* 30)	12 weeks	CRP > 3: Final HDRS: 5.20, 6.53 (SD) CRP < 3: Final HDRS: 4.67, s.d. = 4.33	CRP > 3: Final HDRS: 7.33, s.d. = 7.84 CRP < 3: Final HDRS: 5.77, s.d. = 5.72
Ellegaard et al (2019)^87^	80	80	None/No	Bipolar disorders (1 or 2 current episode depressed MADRS > 17), DSM-IV	TAU + NAC 1500 g b.i.d. (*n =* 40) *v*. TAU + placebo b.i.d. (*n =* 40)	20 weeks	Final MADRS: 16.3, s.d. = 2.3	Final MADRS: 15.6, s.d. = 2.4
Berk et al (2019)^86^	181	148	None, No	Bipolar disorders (1 or 2 current MDE), DSM-IV	TAU + NAC 2000 mg o.d. (*n* = 59) *v*. TAU + NAC 2000 mg o.d. + combination nutraceutical treatment (*n* = 61) *v*. TAU + placebo (*n =* 61)	16 weeks	MADRS change: NAC: –14.2, s.e. = 1.7 combination nutraceutical treatment: –13.2, s.e. = 1.8	MADRS change: –12.9, s.e. = 1.7

MADRS, Montgomery–Åsberg Depression Rating Scale; LSM, least squares mean; NOS, not otherwise specified; MDE, major depressive episode; TAU, treatment as usual; b.i.d., twice daily; ASA, aspirin; CRP, C-reactive protein; NR, not reported.

### Use of immunomodulatory agents for the treatment of MDD

In the first published RCT of the selective cyclooxygenase (COX)-2 inhibitor celecoxib for the treatment of MDD, celecoxib (in addition to reboxetine) was more efficacious than placebo in reducing depressive symptoms.^59^ The reduction in scores on the Hamilton Rating Scale for Depression (HRSD) from baseline to end of trial was 55% in the celecoxib/reboxetine group compared with 33% in the placebo group; 75% of the participants in the celecoxib/reboxetine group were responders compared with 45% in the placebo group.^55^ Following this initial encouraging pilot study, there have been favourable results in trials of celecoxib in MDD as an augmenting agent to sertraline and fluoxetine.^60–62^

Two studies assessed celecoxib as an augmenting agent to sertraline; both reported significantly greater changes in HRSD (mean difference  3.35 (95% CI 1.08–5.61), *t*(38) = 2.99, *P* = 0.005 and (13.7 (s.d. = 3.8) *v*. −8.8 (s.d. = 4.5)) in the celecoxib group compared with placebo group.^61,62^ The third compared celecoxib with fluoxetine and reported a significant difference between the two treatments at the end-point (week 6) (*t* = 3.35, d.f. = 38, *P* = 0.001), also reporting a significant group × time interaction.^60^

Further candidate inflammatory targets for treatment of MDD would include inflammatory cytokines, adhesion molecules and cellular components of the inflammatory response.^63^ Cytokine inhibitors (for example infliximab) target inflammatory cytokines such as TNF-α, IL-1, IL-6R, and IL-12/23 and the cell adhesion molecule α4-integrin. They are approved by the US Food and Drug Administration and recommended by the UK National Institute for Health and Care Excellence for the treatment of autoimmune disorders including rheumatoid arthritis, ulcerative colitis, Crohn disease, psoriasis, multiple sclerosis and ankylosing spondylitis. A recent systematic review of these cytokine inhibitors in patients without a diagnosis of MDD showed that these drugs lead to an overall improvement in depressive symptoms compared with placebo, independent of their effect on physical health.^26^ In RCT in patients with treatment-resistant MDD, there was no overall difference between infliximab and placebo, even when controlling for baseline level of inflammation.^64^ A phase II trial of an anti-IL-6 mAb (sirukumab) in patients with MDD and elevated plasma CRP (ClinicalTrials.gov Identifier: NCT02473289) is awaiting publication.

Another candidate immunomodulatory agent for the treatment of MDD is the anti-inflammatory tetracycline antibiotic minocycline. This pleiotropic agent is postulated to have antidepressant action by inhibiting neurotoxic factors (i.e. proinflammatory cytokines, reactive oxygen species) released by activated microglia, and by inducing neuroprotective factors (i.e. anti-inflammatory cytokines, antioxidants, neurotrophic factors) released by astrocytes.^65^ Minocycline has been studied as a monotherapy or an augmenting agent in patients with MDD. As a monotherapy, it was shown to have clinically significant improvements in depressive symptoms compared with placebo in patients with HIV and comorbid MDD.^66^

Three clinical trials have evaluated minocycline as an augmenting agent in MDD: two (one open-label study, one RCT) reported significant reductions in depressive symptoms with minocycline;^67,68^ the third one, an RCT in 41 patients with treatment-resistant MDD, reported a mean change in HRSD scores of over 18 points in the minocycline group, compared with −0.2 in the placebo group (effect size −1.21, *P* < 0.001).^68^ Given the large effect size and small sample size, this study is now being replicated in a larger sample (ClinicalTrials.gov Identifier: NCT03947827).

In a separate RCT of minocycline in 71 patients with MDD (not necessarily treatment resistant), the investigators reported no significant difference in the reduction on the Montgomery–Åsberg Depression Rating Scale (MADRS) across the 12 weeks of treatment between the minocycline and placebo groups, effect size 0.46 (95% CI −7.1 to 3.2), *P* = 0.02. However, there was a significant improvement in psychosocial function in the minocycline group.^69^ In a recent meta-analysis, the antidepressant effect of minocycline had an overall moderate and statistically significant effect size (−0.78, 95% CI −0.4 to −1.33, *P* = 0.005).^70^

NAC is an over-the-counter supplement that has peripheral and central immunomodulatory effects. NAC has been studied across multiple psychiatric diagnoses,^71^ appearing safe and effective for schizophrenia, but lacking consistent evidence of efficacy in MDD and bipolar disorders. For MDD, one published RCT in a reasonably large sample (*n* = 252) showed that adjunctive NAC was not efficacious in reducing depressive symptoms compared with placebo.^72^ More recently, a smaller study (*n* = 57) compared NAC versus placebo in patients with MDD or bipolar disorders, with evidence of inflammation at baseline (CRP>3 mg/L) or low baseline inflammation (CRP < 3 mg/L).^73^ Those with high baseline inflammation had a significantly greater reduction in depressive symptoms with NAC than with placebo but the analysis did not separate those with MDD or bipolar disorders (*P* = 0.04).^73^ These findings suggest that NAC may be effective in patients who exhibit an inflammatory biotype.

A recent meta-analysis evaluated the efficacy and safety of anti-inflammatory agents as monotherapy or adjunctive treatment for MDD.^74^ The drugs included in this review were celecoxib, eicosapentaenoic acid (EPA), docosahexaenoic acid (DHA), lovastatin, atorvastatin, simvastatin, minocycline, pioglitazone, modafinil and NAC. Overall, in this analysis of 26 RCTs, anti-inflammatory agents reduced depressive symptoms (SMD = −0.55, 95% CI −0.75 to −0.35, *I*^2^ = 71%) and were associated with higher response (relative risk (RR) = 1.52, 95% CI 1.30 to 1.79, *I*^2^ = 29%) and remission rates (RR = 1.79, 95% CI 1.29 to 2.49, *I*^2^ = 41%) compared with placebo.^74^ Anti-inflammatory agents were shown to be safe, with the only significant difference between them and placebo being the incidence of gastrointestinal adverse events.^74^ The authors asserted that, based on the evidence of their analysis, anti-inflammatory agents are effective and relatively safe treatment options for MDD. However, some key limitations detract from this conclusion. The authors included medications that do not have direct anti-inflammatory effects: omega-3, pioglitazone, statins, and modafinil (which has therapeutic mechanisms similar to traditional monoaminergic antidepressants). They also did not include a number of studies that appeared to have met their inclusion criteria and that have been included in previous meta-analyses.^10,75^ Moreover, although the safety of these medications was reported as favourable, adverse events were poorly reported in the original studies.^75^

The latter comment regarding safety is salient as the literature would suggest safety is yet to be reliably established, particularly with the short duration of most trials.^75^ NSAIDs are well documented to have more concerning side-effects than traditional antidepressants, specifically gastric bleeding and impaired renal function. Further, celecoxib is known to increase the risks of cardiovascular events.^10^ This risk is not completely understood in the context of MDD and bipolar disorders, which are both conditions associated with an independent risk for cardiovascular disease.^41,76,77^ With respect to the cytokine inhibitors, these have been associated with opportunistic infections such as tuberculosis because of immunosuppression.^78^ Given the high risks of immunosuppression and adverse side-effects with these agents, it is important to proceed cautiously when designing clinical trials in patients with mood disorders especially given the high degree of physical comorbidity in this population.^79,80^

### Use of immunomodulatory agents for the treatment of bipolar disorder

Open-label studies of minocycline have shown significant improvements in depressive symptoms in patients with bipolar disorder depression.^81,82^ A recent meta-analysis of eight published RCTs of immunomodulatory agents in bipolar disorder depression showed an overall effect size of −0.40 (95% CI −0.14 to −0.65, *P* = 0.002), suggesting a moderate antidepressant effect with good overall tolerability.^12^ Included drugs were DHA/EPA, celecoxib, aspirin, pioglitazone and NAC. Subgroup analysis of each class of drug revealed non-significant effect sizes, with the exception of NAC. This analysis was limited by the dearth of studies in bipolar disorders limiting the power.

Since the publication of this meta-analysis, a relatively large (*n* = 99) 2 × 2 factorial design trial of minocycline and aspirin showed no main effect for either treatment, although participants with elevated IL-6 appeared to have a more favourable response to adjunctive minocycline compared with placebo.^83^ Future studies are needed to assess the efficacy and safety of minocycline in treating bipolar depression; we are aware of at least one large study currently underway to assess the efficacy of both minocycline and celecoxib for bipolar depression.^84^

An early study of NAC in bipolar depression was promising with NAC treatment leading to a significant improvement on the MADRS (least squares mean difference –8.05 (95% CI –13.16 to 2.95), *P* = 0.002) compared with placebo.^85^ However, a meta-analysis that included two RCTs in bipolar disorders found there was no significant group differences in antidepressant effects (*n* = 124, SMD = −0.59, 95% CI −1.48 to 0.3, *I^2^* = 83% *P* = 0.19).^71^

Subsequently there have been several studies assessing its efficacy in treating bipolar disorder depression. The largest of these studies was in 148 patients with bipolar disorder depression who were treated adjunctively with either NAC, NAC plus a nutraceutical agent that may increase mitochondrial biogenesis or placebo.^86^ The analysis of the primary outcome was negative, with no difference in change in depressive symptoms among the three groups. However, there was a significant improved delayed response (20 weeks post-discontinuation) in the NAC plus nutraceutical group.^86^ In another study of the efficacy of adjunctive NAC in 80 patients with bipolar disorder acute depression, NAC was not superior to placebo.^87^

However, in a recent study comparing NAC, aspirin, NAC plus aspirin and placebo, at 16 weeks,

NAC plus aspirin was associated with a higher rate of response (67%), than NAC alone (57%), placebo (55%) or aspirin (33%) alone.^88^

To our knowledge only one RCT has prospectively stratified patients based on evidence of inflammation. A recent 12-week RCT evaluated the effects of adjunctive intravenous infliximab for the treatment of bipolar depression in patients with biochemical (i.e. elevated CRP ≥ 5 mg/L) or phenotypical (i.e. obesity, diabetes type 1 or 2, inflammatory bowel disease, rheumatological disorder, daily cigarette smoking, or migraine headaches) evidence of inflammation. Despite this stratification, infliximab showed no reduction in overall depressive symptoms compared with placebo.^89^ The authors did, however, report an association between early childhood adversity and response to infliximab, suggesting a possible subgroup of patients who could benefit from this form of treatment.

There is some evidence from a few small studies suggesting that immunomodulatory medications are beneficial in mania. In a recent meta-analysis of three RCTs of celecoxib, aspirin and NAC, respectively, the overall effect size for treatment of manic symptoms was −0.72 (95% CI −1.31 to −0.13, *P* = 0.02).^10^

In a secondary analysis of a large study of 482 patients with bipolar disorders receiving either lithium or quetiapine, the trajectory of either depressive or manic symptoms did not significantly differ in patients taking concomitant NSAIDs acetaminophen (*n* = 177) and, those who did not.^90^ However, anti-inflammatory drugs in this study were not the study medication and were only self-reported by patients. Given the small number of reliable studies and their small samples, one cannot draw firm conclusions from their findings, although they suggest that further, well-designed trials investigating the antimanic effects of immunomodulatory agents are warranted.

Overall, although there are some small promising studies in bipolar disorders, results from recent large replication studies have been negative. Some *post hoc* analyses suggest that immunomodulatory agents may be effective for a subset of patients (for example those with elevated IL-6 or those with a history of childhood adversity). However, further work is required to confirm these findings and establish whether some clinical or biological phenotypes may be more responsive to these drugs.

## Discussion

### Main findings

This critical review highlights converging evidence for a bidirectional relationship between an activated inflammatory response and the onset and persistence of mood symptoms in a subset of patients. However, few prospective studies have been conducted thus far and using this approach could provide vital insight into a putative causal link between inflammation and mood disorders. Current evidence suggests that an activated inflammatory response is associated with treatment resistance and may predict poor response to traditional antidepressant medications.^91,92^ However, results from clinical trials of immunomodulatory agents in mood disorders have been conflicting and the efficacy of these drugs is yet to be established.

### Interpretation of our findings

One potential explanation for the inconsistent findings from RCTs of immunomodulatory agents is that these treatments have not been targeted towards an ‘inflamed’ subset of patients. Very few studies included in this review stratified treatment based on *a priori* markers of an activated immune response. Only about 30% of individuals with MDD show peripheral evidence of inflammation and immunomodulatory agents are only likely to benefit this specific subset of individuals.^30^ Nonetheless, results from the recent study of infliximab for bipolar depression has shown that stratifying patients based on peripheral markers of inflammation such as CRP or phenotypic evidence of inflammation does not necessarily confer benefit from immunomodulatory agents.^89^

### Choice of candidate peripheral biomarkers

A major gap in the current evidence relates to the choice of candidate biomarkers that could be utilised to stratify patients to an ‘inflamed’ subtype. Historically, when utilised, studies have used individual peripheral inflammatory markers such as CRP, IL-6 or TNF-α. This approach is limited as acute phase proteins and inflammatory cytokines are highly influenced by multiple factors including diet, BMI and smoking status. Moreover, each individual marker may have varying degrees of inflammatory versus anti-inflammatory properties.^36,37^ Recent studies suggest that using composite measures may be a more suitable approach. One such study developed a multisystem data-driven composite inflammatory biomarker, using *POLG*, *ADARB1*, *OGG*, 8-oxoGuo, leucocytes and age, to distinguish patients with bipolar disorders from healthy controls with a sensitivity of 73% and specificity of 71%.^93^

Utilising computer generated binary clustering, another study was able to stratify patients with MDD to ‘inflamed’ and ‘uninflamed’ subtypes using peripheral immune cell counts. The inflamed subgroup had increased monocyte, CD4^+^, neutrophil counts, CRP and IL-6. This subgroup also had more severe depressive symptoms.^94^ Furthermore, there is evidence that symptom clusters of depression may contribute to the ‘inflamed’ subtype; for example anhedonia has been associated with increased CRP and atypical depressive symptoms (increased appetite, weight gain) have been associated with increased CRP, IL-1RA and IL-6.^95,96^

### Combining peripheral and central markers of inflammation

To date, most studies have focused solely on peripheral markers of inflammation and a more informative approach may be to assess these alongside sophisticated neuroimaging techniques such as PET imaging or more invasive measures of central inflammation, for example in cerebrospinal fluid. This would allow the assessment of the correlation between the central markers of inflammation and the more pragmatic peripheral markers. For instance, a recent study, utilising a composite approach, measured the blood serum concentration of several products synthesised by activated microglia and to some extent astroglia – for example prostaglandin E_2_ (PGE_2_), prostaglandin F_2_ alpha (PGF_2α_), and TNF-α – and controlled by CRP. This study showed that ln(PGE_2_/CRP) and ln(TNF-α/CRP) consistently correlated with TSPO V_T_ (and hence microglial activation and neuroinflammation) in patients with MDD.^97^ If this approach leads to the identification of peripheral markers that are surrogate markers of neuroinflammation, they can then be used in future RCTs of immunomodulatory therapeutics. Taken together, utilising peripheral and central markers of inflammation and targeting specific symptom clusters in patients with mood disorders may provide more consistent results in clinical trials of repurposed or novel immunomodulatory drugs.

### Addressing the heterogeneity of mood disorders

Finally, it is also important to consider the heterogeneity of mood disorders as an ongoing challenge in designing RCTs of any novel intervention. Using the Research Domain Criteria approach to examine specific symptom subsets (for example anhedonia, motivation, suicidal ideation) rather than solely relying on traditional assessment scales, may be a more sensitive way to assess the efficacy of immunomodulatory agents. Future trials should use the existing evidence on inflammatory biomarkers to recruit and stratify participants who may be more likely to respond to repurposed or novel immunomodulatory medications. Although further trials of these agents remain warranted, they must incorporate enriched patient samples and mechanistic evaluations into their design. Otherwise, the promise of translating the use of these agents to the clinic is likely to remain unfulfilled.

## References

[1] World Health Organization. WHO Global Burden of Disease (2008): 2004 Update. WHO, 2008.

[2] World Health Organization. Depression and Other Common Mental Disorders: Global Health Estimates. WHO, 2017.

[3] Greenberg PE, Kessler RC, Birnbaum HG, Leong SA, Lowe SW, Berglund PA, The economic burden of depression in the United States: how did it change between 1990 and 2000? J Clin Psychiatry 2003 Dec; 64: 1465–75. 14728109 10.4088/jcp.v64n1211

[4] Strawbridge R, Young AH, Cleare AJ. Biomarkers for depression: recent insights, current challenges and future prospects. Neuropsychiatr Dis Treat 2017; 13: 1245–62. 28546750 10.2147/NDT.S114542 PMC5436791

[5] Insel T, Cuthbert B, Garvey M, Heinssen R, Pine DS, Quinn K, Research Domain Criteria (RDoC): toward a new classification framework for research on mental disorders. Am J Psychiatry 2010; 167: 748–51. 20595427 10.1176/appi.ajp.2010.09091379

[6] Rosenblat JD, McIntyre RS, Alves GS, Fountoulakis KN, Carvalho AF. Beyond monoaminesnovel targets for treatment-resistant depression: a comprehensive review. Curr Neuropharmacol 2015; 13: 636–55. 26467412 10.2174/1570159X13666150630175044 PMC4761634

[7] Rizvi SJ, Grima E, Tan M, Rotzinger S, Lin P, McIntyre RS, Treatment-resistant depression in primary care across Canada. Can J Psychiatry 2014; 59: 349–57. 25007419 10.1177/070674371405900702 PMC4086317

[8] Macaluso M, Preskorn SH. Antidepressants: From Biogenic Amines to New Mechanisms of Action. Springer, 2018.

[9] Thomas L, Kessler D, Campbell J, Morrison J, Peters TJ, Williams C, Prevalence of treatment-resistant depression in primary care: cross-sectional data. Br J Gen Pract 2013; 63: e852–8. 24351501 10.3399/bjgp13X675430 PMC3839394

[10] Husain MI, Strawbridge R, Stokes PRA, Young AH. Anti-inflammatory treatments for mood disorders: Systematic review and meta-analysis. J Psychopharmacol 2017; 31: 1137–48. 28858537 10.1177/0269881117725711

[11] Rosenblat JD, McIntyre RS. Bipolar Disorder and Immune Dysfunction: Epidemiological Findings, Proposed Pathophysiology and Clinical Implications. Vol. 7, Brain Sciences. MDPI AG; 2017. 10.3390/brainsci7110144 PMC5704151 29084144

[12] Rosenblat JD, Kakar R, Berk M, Kessing LV, Vinberg M, Baune BT, Anti-inflammatory agents in the treatment of bipolar depression: a systematic review and meta-analysis. Bipolar Disord 2016; 18: 89–101. 26990051 10.1111/bdi.12373

[13] Ayorech Z, Tracy DK, Baumeister D, Giaroli G. Taking the fuel out of the fire: evidence for the use of anti-inflammatory agents in the treatment of bipolar disorders. J Affect Disord 2015; 174: 467–78. 25553408 10.1016/j.jad.2014.12.015

[14] Fond G, Hamdani N, Kapczinski F, Boukouaci W, Drancourt N, Dargel A, Effectiveness and tolerance of anti-inflammatory drugs’ add-on therapy in major mental disorders: a systematic qualitative review. Acta Psychiatr Scand 2014; 129: 163–79. 24215721 10.1111/acps.12211

[15] Faridhosseini F, Sadeghi R, Farid L, Pourgholami M. Celecoxib: a new augmentation strategy for depressive mood episodes. A systematic review and meta-analysis of randomized placebo controlled trials. Hum Psychopharmacol 2014; 29: 216–23. 24911574 10.1002/hup.2401

[16] Köhler O, Benros M E, Nordentoft M, Farkouh ME, Iyengar RL, Mors O, Effect of antiinflammatory treatment on depression, depressive symptoms, and adverse effects a systematic review and meta-analysis of randomized clinical trials. JAMA Psychiatry 2014; 71: 1381–91. 25322082 10.1001/jamapsychiatry.2014.1611

[17] Roman M, Irwin MR. Novel neuroimmunologic therapeutics in depression: a clinical perspective on what we know so far. Brain Behav Immun 2020; 83: 7–21. 31550500 10.1016/j.bbi.2019.09.016 PMC6940145

[18] World Health Organization. The ICD-10 Classification of Mental and Behavioural Disorders Clinical Descriptions and Diagnostic Guidelines. WHO, 1992 (http://www.who.int/classifications/icd/en/bluebook.pdf).

[19] First M, Gibbon M, Spitzer R, Williams J. User Guide for the Structural Clinical Interview for DSM-IV Axis I Disorders. APA. American Psychiatric Association, 1996.

[20] American Psychiatric Association. Diagnostic and Statistical Manual of Mental Disorders (DSM-5). American Psychiatric Association, 2013.

[21] Raju TN. The noble chronicles. Lancet 1998; 352: 661. 10.1016/s0140-6736(05)60452-7 9863832

[22] Hart BL. Biological basis of the behavior of sick animals. Neurosci Biobehav Rev 1988; 12: 123–37. 3050629 10.1016/s0149-7634(88)80004-6

[23] Smith RS. The macrophage theory of depression. Med Hypotheses 1991; 35: 298–306. 1943879 10.1016/0306-9877(91)90272-z

[24] Capuron L, Miller AH. Immune system to brain signaling: neuropsychopharmacological implications. Pharmacol Ther 2011; 130: 226–38. 21334376 10.1016/j.pharmthera.2011.01.014 PMC3072299

[25] Mardini HE, Kip KE, Wilson JW. Crohn's disease: a two-year prospective study of the association between psychological distress and disease activity. Dig Dis Sci 2004 Mar; 49: 492–7. 15139504 10.1023/b:ddas.0000020509.23162.cc

[26] Kappelmann N, Lewis G, Dantzer R, Jones PB, Khandaker GM. Antidepressant activity of anticytokine treatment: a systematic review and meta-analysis of clinical trials of chronic inflammatory conditions. Mol Psychiatry 2018; 23: 335–43. 27752078 10.1038/mp.2016.167 PMC5794896

[27] Cipriani A, Furukawa TA, Salanti G, Chaimani A, Atkinson LZ, Ogawa Y, Comparative efficacy and acceptability of 21 antidepressant drugs for the acute treatment of adults with major depressive disorder: a systematic review and network meta-analysis. Lancet 2018; 391: 1357–66. 29477251 10.1016/S0140-6736(17)32802-7 PMC5889788

[28] Strawbridge R, Carter B, Marwood L, Bandelow B, Tsapekos D, Nikolova VL, Augmentation therapies for treatment-resistant depression: systematic review and meta-analysis. Br J Psychiatry 2019; 214: 42–51. 30457075 10.1192/bjp.2018.233

[29] Köhler CA, Freitas TH, Maes M, de Andrade NQ, Liu CS, Fernandes BS, Peripheral cytokine and chemokine alterations in depression: a meta-analysis of 82 studies. Acta Psychiatr Scand 2017; 135: 373–87. 28122130 10.1111/acps.12698

[30] Osimo EF, Baxter LJ, Lewis G, Jones PB, Khandaker GM. Prevalence of low-grade inflammation in depression: a systematic review and meta-Analysis of CRP levels. Psychol Med 2019; 49: 1958–70. 31258105 10.1017/S0033291719001454 PMC6712955

[31] Köhler CA, Freitas TH, Stubbs B, Maes M, Solmi M, Veronese N, Peripheral alterations in cytokine and chemokine levels after antidepressant drug treatment for major depressive disorder: systematic review and meta-analysis. Mol Neurobiol 2018; 55: 4195–206. 28612257 10.1007/s12035-017-0632-1

[32] Liu JJ, Wei YB, Strawbridge R, Bao Y, Chang S, Shi L, Peripheral cytokine levels and response to antidepressant treatment in depression: a systematic review and meta-analysis. Mol Psychiatry 2020; 25: 339–50. 31427752 10.1038/s41380-019-0474-5

[33] Grosse L, Carvalho LA, Birkenhager TK, Hoogendijk WJ, Kushner SA, Drexhage HA, Circulating cytotoxic T cells and natural killer cells as potential predictors for antidepressant response in melancholic depression. Restoration of T regulatory cell populations after antidepressant therapy. Psychopharmacology (Berl) 2016; 233: 1679–88. 25953327 10.1007/s00213-015-3943-9

[34] Chu AL, Stochl J, Lewis G, Zammit S, Jones PB, Khandaker GM. Longitudinal association between inflammatory markers and specific symptoms of depression in a prospective birth cohort. Brain Behav Immun 2019; 76: 74–81. 30414442 10.1016/j.bbi.2018.11.007 PMC6363967

[35] Khandaker GM, Stochl J, Zammit S, Goodyer I, Lewis G, Jones PB. Childhood inflammatory markers and intelligence as predictors of subsequent persistent depressive symptoms: a longitudinal cohort study. Psychol Med 2018; 48: 1514–22. 29140226 10.1017/S0033291717003038 PMC6088526

[36] Horn SR, Long MM, Nelson BW, Allen NB, Fisher PA, Byrne ML. Replication and reproducibility issues in the relationship between C-reactive protein and depression: a systematic review and focused meta-analysis. Brain Behav Immun 2018; 73: 85–114. 29928963 10.1016/j.bbi.2018.06.016 PMC6800199

[37] Giudice MD, Gangestad SW. Rethinking IL-6 and CRP: why they are more than inflammatory biomarkers, and why it matters. Brain Behav Immu*n* 2018; 70: 61–75. 29499302 10.1016/j.bbi.2018.02.013

[38] Sayana P, Colpo GD, Simões LR, Giridharan VV, Teixeira AL, Quevedo J, A systemic review of evidence for the role of inflammatory biomarkers in bipolar patients. J Psychiatr Res 2017; 92: 160–82. 28458141 10.1016/j.jpsychires.2017.03.018

[39] Rosenblat JD, Mcintyre RS. Are medical comorbid conditions of bipolar disorder due to immune dysfunction? Acta Psychiatr Scand 2015; 132: 180–91. 25772638 10.1111/acps.12414

[40] Prieto ML, Schenck LA, Kruse JL, Klaas JP, Chamberlain AM, Bobo W V, Long-term risk of myocardial infarction and stroke in bipolar I disorder: a population-based cohort study. J Affect Disord 2016; 194: 120–7. 26820761 10.1016/j.jad.2016.01.015 PMC4909505

[41] Goldstein BI, Carnethon MR, Matthews KA, McIntyre RS, Miller GE, Raghuveer G, Major depressive disorder and bipolar disorder predispose youth to accelerated atherosclerosis and early cardiovascular disease: a scientific statement from the American Heart Association. Circulation 2015; 132: 965–86. 26260736 10.1161/CIR.0000000000000229

[42] Ayerbe L, Forgnone I, Addo J, Siguero A, Gelati S, Ayis S. Hypertension risk and clinical care in patients with bipolar disorder or schizophrenia; a systematic review and meta-analysis. J Affect Disord 2018; 225: 665–70. 28915505 10.1016/j.jad.2017.09.002

[43] SayuriYamagata A, Brietzke E, Rosenblat JD, Kakar R, McIntyre RS. Medical comorbidity in bipolar disorder: the link with metabolic-inflammatory systems. J Affect Disord 2017; 211: 99–106. 28107669 10.1016/j.jad.2016.12.059

[44] Enache D, Pariante CM, Mondelli V. Markers of central inflammation in major depressive disorder: a systematic review and meta-analysis of studies examining cerebrospinal fluid, positron emission tomography and post-mortem brain tissue. Brain Behav Immun 2019; 81: 24–40. 31195092 10.1016/j.bbi.2019.06.015

[45] Giridharan VV, Sayana P, Pinjari OF, Ahmad N, da Rosa MI, Quevedo J, Postmortem evidence of brain inflammatory markers in bipolar disorder: a systematic review. Mol Psychiatry 2020; 25: 94–113. 31249382 10.1038/s41380-019-0448-7

[46] Haarman BCMB, Riemersma-Van der Lek RF, de Groot JC, Ruhé HGE, Klein HC, Zandstra TE, Neuroinflammation in bipolar disorder - A [11C]-(R)-PK11195 positron emission tomography study. Brain Behav Immun 2014; 40: 219–25. 24703991 10.1016/j.bbi.2014.03.016

[47] Narayanaswami V, Dahl K, Bernard-Gauthier V, Josephson L, Cumming P, Vasdev N. Emerging PET radiotracers and targets for imaging of neuroinflammation in neurodegenerative diseases: outlook beyond TSPO. Mol Imaging 2018; 17: 1536012118792317. 30203712 10.1177/1536012118792317 PMC6134492

[48] Miller AH, Raison CL. The role of inflammation in depression: from evolutionary imperative to modern treatment target. Nat Rev Immunology 2016; 16: 22–34. 26711676 10.1038/nri.2015.5 PMC5542678

[49] Bin ZC, Lindler KM, Owens AW, Daws LC, Blakely RD, Hewlett WA. Interleukin-1 receptor activation by systemic lipopolysaccharide induces behavioral despair linked to MAPK regulation of CNS serotonin transporters. Neuropsychopharmacology 2010 Dec; 35: 2510–20. 20827273 10.1038/npp.2010.116 PMC3055584

[50] Raison CL, Dantzer R, Kelley KW, Lawson MA, Woolwine BJ, Vogt G, CSF concentrations of brain tryptophan and kynurenines during immune stimulation with IFN-alpha: relationship to CNS immune responses and depression. Mol Psychiatry 2010; 15: 393–403. 19918244 10.1038/mp.2009.116 PMC2844942

[51] Maes M, Leonard BE, Myint a, Kubera M, Verkerk R. The new “5-HT” hypothesis of depression. Prog Neuro-Psychopharmacology Biol Psychiatry 2011; 35: 702–21. 10.1016/j.pnpbp.2010.12.017 21185346

[52] Tavares RG, Tasca CI, Santos CES, Alves LB, Porciúncula LO, Emanuelli T, Quinolinic acid stimulates synaptosomal glutamate release and inhibits glutamate uptake into astrocytes. Neurochem Int 2002; 40: 621–7. 11900857 10.1016/s0197-0186(01)00133-4

[53] Tilleux S, Hermans E. Neuroinflammation and regulation of glial glutamate uptake in neurological disorders. J Neurosci Res 2007; 85: 2059–70. 17497670 10.1002/jnr.21325

[54] Hardingham GE, Fukunaga Y, Bading H. Extrasynaptic NMDARs oppose synaptic NMDARs by triggering CREB shut-off and cell death pathways. Nat Neurosci 2002; 5: 405–14. 11953750 10.1038/nn835

[55] Berk M, Kapczinski F, Andreazza A, Dean O, Giorlando F, Maes M, Pathways underlying neuroprogression in bipolar disorder: focus on inflammation, oxidative stress and neurotrophic factors. Neurosci Biobehav Rev 2011; 35: 804–17. 20934453 10.1016/j.neubiorev.2010.10.001

[56] Rees JN, Florang VR, Anderson DG, Doom JA. Lipid peroxidation products inhibit dopamine catabolism yielding aberrant levels of a reactive intermediate. Chem Res Toxicol 2007; 20: 1536–42. 17887726 10.1021/tx700248y

[57] Green K, Brand MD, Murphy MP. Prevention of mitochondrial oxidative damage as a therapeutic strategy in diabetes. Diabetes 2004; 53(Suppl 1): S110–8. 14749275 10.2337/diabetes.53.2007.s110

[58] Zuo D-Y, Wu Y-L, Yao W-X, Cao Y, Wu C-F, Tanaka M. Effect of MK-801 and ketamine on hydroxyl radical generation in the posterior cingulate and retrosplenial cortex of free-moving mice, as determined by in vivo microdialysis. Pharmacol Biochem Behav 2007; 86: 1–7. 16806445 10.1016/j.pbb.2006.05.010

[59] Müller N, Schwarz MJ, Dehning S, Douhe A, Cerovecki A, Goldstein-Müller B, The cyclooxygenase-2 inhibitor celecoxib has therapeutic effects in major depression: results of a double-blind, randomized, placebo controlled, add-on pilot study to reboxetine. Mol Psychiatry 2006; 11: 680–4. 16491133 10.1038/sj.mp.4001805

[60] Akhondzadeh S, Jafari S, Raisi F, Nasehi AA, Ghoreishi A, Salehi B, Clinical trial of adjunctive celecoxib treatment in patients with major depression: a double blind and placebo controlled trial. Depress Anxiety 2009; 26: 607–11. 19496103 10.1002/da.20589

[61] Majd M, Hashemian F, Hosseinib SM, Shariatpanahi MV, Sharifid A. A randomized, doubleblind, placebo-controlled trial of celecoxib augmentation of sertraline in treatment of drug-naive depressed women: a pilot study. Iran J Pharm Res 2015; 14: 891–9. 26330878 PMC4518118

[62] Abbasi SH, Hosseini F, Modabbernia A, Ashrafi M, Akhondzadeh S. Effect of celecoxib add-on treatment on symptoms and serum IL-6 concentrations in patients with major depressive disorder: Randomized double-blind placebo-controlled study. J Affect Disord 2012; 141: 308–14. 22516310 10.1016/j.jad.2012.03.033

[63] Miller AH, Haroon E, Felger JC. Therapeutic implications of brain-immune interactions: treatment in translation. Neuropsychopharmacology 2017; 42: 334–59. 27555382 10.1038/npp.2016.167 PMC5143492

[64] Raison CL, Rutherford RE, Woolwine BJ, Shuo C, Schettler P, Drake DF, A randomized controlled trial of the tumor necrosis factor antagonist infliximab for treatment-resistant depression: the role of baseline inflammatory biomarkers. JAMA Psychiatry 2013; 70: 31–41. 22945416 10.1001/2013.jamapsychiatry.4 PMC4015348

[65] Soczynska JK, Mansur RB, Brietzke E, Swardfager W, Kennedy SH, Woldeyohannes HO, Novel therapeutic targets in depression: minocycline as a candidate treatment. Behav Brain Res 2012; 235: 302–17. 22963995 10.1016/j.bbr.2012.07.026

[66] Emadi-Kouchak H, Mohammadinejad P, Asadollahi-Amin A, Rasoulinejad M, Zeinoddini A, Yalda A, Therapeutic effects of minocycline on mild-to-moderate depression in HIV patients: A double-blind, placebo-controlled, randomized trial. Int Clin Psychopharmacol 2016; 31: 20–6. 26465919 10.1097/YIC.0000000000000098

[67] Miyaoka T, Wake R, Furuya M, Liaury K, Ieda M, Kawakami K, Minocycline as adjunctive therapy for patients with unipolar psychotic depression: an open-label study. Prog Neuro-Psychopharmacol Biol Psychiatry 2012; 37: 222–6. 10.1016/j.pnpbp.2012.02.002 22349578

[68] Husain MI, Chaudhry IB, Husain N, Khoso AB, Rahman RR, Hamirani MM, Minocycline as an adjunct for treatment-resistant depressive symptoms: a pilot randomised placebo-controlled trial. J Psychopharmacol 2017; 31: 1166–75. 28857658 10.1177/0269881117724352

[69] Dean OM, Kanchanatawan B, Ashton M, Mohebbi M, Ng CH, Maes M, Adjunctive minocycline treatment for major depressive disorder: a proof of concept trial. Aust N Z J Psychiatry 2017; 51: 829–40. 28578592 10.1177/0004867417709357

[70] Rosenblat JD, McIntyre RS. Efficacy and tolerability of minocycline for depression: a systematic review and meta-analysis of clinical trials. J Affect Disord 2018; 227: 219–25. 29102836 10.1016/j.jad.2017.10.042

[71] Zheng W, Zhang Q-E, Cai D-B, Yang X-H, Qiu Y, Ungvari GS, N -acetylcysteine for major mental disorders: a systematic review and meta-analysis of randomized controlled trials. Acta Psychiatr Scand 2018; 137: 391–400. 29457216 10.1111/acps.12862

[72] Berk M, Dean OM, Cotton SM, Jeavons S, Tanious M, Kohlmann K, The efficacy of adjunctive N-acetylcysteine in major depressive disorder. J Clin Psychiatry 2014; 75: 628–36. 25004186 10.4088/JCP.13m08454

[73] Porcu M, Urbano MR, Verri WA, Sabbatini Barbosa D, Baracat M, Vargas HO, Effects of adjunctive N-acetylcysteine on depressive symptoms: modulation by baseline high-sensitivity C reactive protein. Psychiatry Res 2018; 263: 268–74. 29605103 10.1016/j.psychres.2018.02.056

[74] Bai S, Guo W, Feng Y, Deng H, Li G, Nie H, Efficacy and safety of anti-inflammatory agents for the treatment of major depressive disorder: a systematic review and meta-analysis of randomised controlled trials. J Neurol Neurosurg Psychiatry 2020; 91: 21–32. 31658959 10.1136/jnnp-2019-320912

[75] Köhler-Forsberg O, N. Lydholm C, Hjorthøj C, Nordentoft M, Mors O, Benros ME. Efficacy of anti-inflammatory treatment on major depressive disorder or depressive symptoms: meta-analysis of clinical trials. Acta Psychiatr Scand 2019; 139: 404–19. 30834514 10.1111/acps.13016

[76] Goldstein BI, Schaffer A, Wang S, Blanco C. Excessive and premature new-onset cardiovascular disease among adults with bipolar disorder in the US NESARC cohort. J Clin Psychiatry 2015; 76: 163–9. 25742203 10.4088/JCP.14m09300

[77] Penninx BWJH. Depression and cardiovascular disease: epidemiological evidence on their linking mechanisms. Neurosci Biobehav Rev 2017; 74: 277–86. 27461915 10.1016/j.neubiorev.2016.07.003

[78] Cao BL, Qasem A, Sharp RC, Abdelli LS, Naser SA. Systematic review and meta-analysis on the association of tuberculosis in Crohn's disease patients treated with tumor necrosis factor-α inhibitors (Anti-TNFα). World J Gastroenterol 2018; 24: 2764–75. 29991880 10.3748/wjg.v24.i25.2764 PMC6034143

[79] Kopylov U, Vutcovici M, Kezouh A, Seidman E, Bitton A, Afif W. Risk of lymphoma, colorectal and skin cancer in patients with IBD treated with immunomodulators and biologics: a Quebec claims database study. Inflamm Bowel Dis 2015; 21: 1847–53. 25993693 10.1097/MIB.0000000000000457

[80] Singh JA, Cameron C, Noorbaloochi S, Cullis T, Tucker M, Christensen R, Risk of serious infection in biological treatment of patients with rheumatoid arthritis: A systematic review and meta-analysis. Lancet 2015; 386: 258–65. 25975452 10.1016/S0140-6736(14)61704-9 PMC4580232

[81] Soczynska JK, Kennedy SH, Alsuwaidan M, Mansur RB, Li M, McAndrews MP, A pilot, open-label, 8-week study evaluating the efficacy, safety and tolerability of adjunctive minocycline for the treatment of bipolar I/II depression. Bipolar Disord 2017; 19: 198–213. 28599348 10.1111/bdi.12496

[82] Murrough JW, Huryk KM, Mao X, Iacoviello B, Collins K, Nierenberg AA, A pilot study of minocycline for the treatment of bipolar depression: effects on cortical glutathione and oxidative stress in vivo. J Affect Disord 2018; 230: 56–64. 29407539 10.1016/j.jad.2017.12.067

[83] Savitz JB, Teague TK, Misaki M, Macaluso M, Wurfel BE, Meyer M, Treatment of bipolar depression with minocycline and/or aspirin: an adaptive, 2×2 double-blind, randomized, placebo controlled, phase IIA clinical trial. Transl Psychiatry 2018; 8: 27. 29362444 10.1038/s41398-017-0073-7 PMC5802452

[84] Husain MI, Chaudhry IB, Hamirani MM, Minhas FA, Kazmi A, Hodsoll J, Minocycline and celecoxib as adjunctive treatments for bipolar depression: a study protocol for a multicenter factorial design randomized controlled trial. Neuropsychiatr Dis Treat 2017; 13: 1–8. 28031712 10.2147/NDT.S115002 PMC5182039

[85] Berk M, Copolov DL, Dean O, Lu K, Jeavons S, Schapkaitz I, N-Acetyl cysteine for depressive symptoms in bipolar disorder–A double-blind randomized placebo-controlled trial. Biol Psychiatry 2008; 64: 468–75. 18534556 10.1016/j.biopsych.2008.04.022

[86] Berk M, Turner A, Malhi GS, Ng C, Cotton SM, Dodd S, A randomised controlled trial of a mitochondrial therapeutic target for bipolar depression: mitochondrial agents, N-acetylcysteine, and placebo. BMC Med 2019; 17: 18. 30678686 10.1186/s12916-019-1257-1 PMC6346513

[87] Ellegaard PK, Licht RW, Nielsen RE, Dean OM, Berk M, Poulsen HE, The efficacy of adjunctive N-acetylcysteine in acute bipolar depression: a randomized placebo-controlled study. J Affect Disord 2019; 245: 1043–51. 30699846 10.1016/j.jad.2018.10.083

[88] Bauer IE, Green C, Colpo GD, Teixeira AL, Selvaraj S, Durkin K, A double-blind, randomized, placebo-controlled study of aspirin and N-acetylcysteine as adjunctive treatments for bipolar depression. J Clin Psychiatry 2019; 80: 1. 10.4088/JCP.18m12200 30549489

[89] McIntyre RS, Subramaniapillai M, Lee Y, Pan Z, Carmona NE, Shekotikhina M, Efficacy of adjunctive infliximab vs placebo in the treatment of adults with bipolar I/II depression: a randomized clinical trial. JAMA Psychiatry 2019; 76: 783–90. 10.1001/jamapsychiatry.2019.0779 PMC6506894 31066887

[90] Köhler-Forsberg O, Sylvia L, Thase M, Calabrese JR, Deckersbach T, Tohen M, Nonsteroidal anti-inflammatory drugs (NSAIDs) and paracetamol do not affect 6-month mood stabilizing treatment outcome among 482 patients with bipolar disorder. Depress Anxiety 2017; 34: 281–90. 28135023 10.1002/da.22601

[91] Chamberlain SR, Cavanagh J, De Boer P, Mondelli V, Jones DNC, Drevets WC, Treatmentresistant depression and peripheral C-reactive protein. Br J Psychiatry 2019; 214: 11–9. 29764522 10.1192/bjp.2018.66 PMC6124647

[92] Arteaga-Henríquez G, Simon MS, Burger B, Weidinger E, Wijkhuijs A, Arolt V, Low-grade inflammation as a predictor of antidepressant and anti-inflammatory therapy response in MDD patients: a systematic review of the literature in combination with an analysis of experimental data collected in the EU-Moodinflame Consortium. Front Psychiatry 2019; 10: 458. 31354538 10.3389/fpsyt.2019.00458 PMC6630191

[93] Munkholm K, Vinberg M, Pedersen BK, Poulsen HE, Ekstrøm CT, Kessing LV. A multisystem composite biomarker as a preliminary diagnostic test in bipolar disorder. Acta Psychiatr Scand 2019; 139: 227–36. 30383306 10.1111/acps.12983

[94] Lynall ME, Turner L, Bhatti J, Cavanagh J, de Boer P, Mondelli V, Peripheral blood cell–stratified subgroups of inflamed depression. Biol Psychiatry [Epub ahead of print] 2 Dec 2019. Available from: 10.1016/j.biopsych.2019.11.017. 32000983

[95] Felger JC, Haroon E, Patel TA, Goldsmith DR, Wommack EC, Woolwine BJ, What does plasma CRP tell us about peripheral and central inflammation in depression? Mol Psychiatry 2020; 25: 1301–11. 29895893 10.1038/s41380-018-0096-3 PMC6291384

[96] Simmons WK, Burrows K, Avery JA, Kerr KL, Taylor A, Bodurka J, Appetite changes reveal depression subgroups with distinct endocrine, metabolic, and immune states. Mol Psychiatry [Epub ahead of print] 13 Jun 2018. Available from: 10.1038/s41380-018-0093-6. PMC6292746 29899546

[97] Attwells S, Setiawan E, Wilson AA, Rusjan PM, Miler L, Xu C, Replicating predictive serum correlates of greater translocator protein distribution volume in brain. Neuropsychopharmacology 2020; 45: 925–31. 31683271 10.1038/s41386-019-0561-y PMC7162884

